# Connecting the Dots: From Teachers’ Perceived Ability to Teach Reading and Their Knowledge of Language and Literacy Concepts to Students’ Reading Growth

**DOI:** 10.3390/bs15101408

**Published:** 2025-10-16

**Authors:** Pamela Guilbault, George K. Georgiou, Joanna Huynh, Tomohiro Inoue

**Affiliations:** 1 Catholic Independent Schools of Nelson Diocese, Kelowna, BC V1W 4M7, Canada; 2 Faculty of Education, University of Alberta, Edmonton, AB T6G 2G5, Canada; 3 Department of Psychology, The Chinese University of Hong Kong, Hong Kong

**Keywords:** differentiated instruction, literacy, professional development, reading, teacher knowledge, self-efficacy

## Abstract

The purpose of this study was two-fold: (a) to examine the joint contribution of teachers’ knowledge of foundational language and literacy concepts and their perceived ability to teach reading to their students’ reading growth, and (b) to examine whether the effects of these factors were mediated by teachers’ perceived ability to differentiate instruction. A total of 32 language arts teachers and their 582 Grade 3 to 9 students (48% female) participated in the study. Teachers completed a survey on their knowledge of phonological awareness, phonics and morphology, and also rated their ability to teach different reading skills and to differentiate reading instruction. Children were assessed at the beginning and end of the school year on the Test of Word Reading Efficiency-2 and on the Test of Silent Reading Efficiency and Comprehension. Results of multilevel modeling indicated that teachers’ knowledge had a direct effect on students’ performance at the end of the school year, even after controlling for students’ earlier reading ability. Teachers’ perceived ability did not predict students’ reading growth either directly or indirectly. Taken together, these findings suggest that we need to invest in increasing teachers’ knowledge around foundational literacy skills.

## 1. Introduction

Teachers are undoubtedly one of the key players in children’s learning (e.g., Hattie, 2023). Since reading is not natural and students must be taught how to “crack” the code of their orthography, one would expect that factors related to teachers should play a crucial role in children’s reading performance. Among the many teacher-related factors that may contribute to children’s reading performance, teachers’ knowledge of different language and literacy concepts as well as their perceived ability to teach different reading-related skills have attracted researchers’ interest. Teachers’ knowledge is an integral component of Desimone’s (2009) professional development model according to which teachers’ professional development would lead to changes in their knowledge, which would then lead to changes in their teaching practices (e.g., differentiation of instruction), and finally lead to changes in student outcomes. Obviously, more knowledgeable teachers are more likely to teach the foundational literacy skills to their students and differentiate instruction. However, beyond possessing adequate knowledge of the foundational literacy skills and how to teach them, researchers indicated that teachers need to also believe in their own ability to teach these skills (e.g., Caprara et al., 2006; Guo et al., 2012). This has been one of the main tenets of Bandura’s (1986) self-efficacy theory, which posits that self-efficacy—the belief in one’s capabilities to execute behaviors necessary for a specific performance—plays a crucial role in motivation, learning, and resilience in educational contexts. Research has shown that when teachers engage in tailored professional development that aligns with their self-efficacy beliefs, their professional practices are positively influenced, leading to enhanced student outcomes (e.g., Kang et al., 2013). Desimone’s (2009) framework supports the notion that coherence between teachers’ professional learning experiences and their self-efficacy beliefs has a more profound and sustained impact on their instructional strategies. Moreover, Bandura’s model highlights that enactive mastery experiences and verbal persuasions during professional development enhance teachers’ self-efficacy and facilitate a deeper engagement with the content they are learning (O’Dea & Harris, 2019).

Despite the theoretical connection between the teachers’ knowledge of language and literacy concepts, perceived ability in teaching reading and students’ reading performance (Desimone, 2009; Desimone & Garet, 2015), only a few studies have examined their association (e.g., Parrila et al., 2024) and they have some important limitations. First, they have all focused on early grades. Obviously, teachers’ knowledge and perceived ability are not relevant only for early grades. In fact, teachers’ knowledge might play an even more important role in later grades as students’ textbooks contain significantly more polymorphemic words (Nippold, 2018) that require teachers to have explicit knowledge of morphology in order to help their students read these words. If they do not have that knowledge, they will be less likely to teach it. Second, only a few studies have examined the role of these factors in students’ reading growth, in other words, after controlling for students’ earlier reading skills. This is important as most previous studies had documented the connection between these variables concurrently and therefore could not shed light on students’ reading growth. Finally, only one study (Parrila et al., 2024) has examined the joint effects of teachers’ knowledge of language and literacy skills and perceived ability to teach these skills on students’ reading growth. As teachers who are more confident also have more knowledge (Podhajski et al., 2009), the effect of these factors on students’ reading growth might be overlapping. Thus, the purpose of this study was to examine the role of both teachers’ knowledge of language and literacy concepts and perceived ability in Grade 3 to 9 students’ reading growth.

### 1.1. Teachers’ Knowledge of Language and Literacy Concepts

Several studies have examined teachers’ knowledge of language and literacy concepts such as phonological awareness, phonics, and morphology (e.g., Bos et al., 2001; Joshi et al., 2009; McMahan et al., 2019; Moats, 1994; Piasta et al., 2020; see also Cunningham et al., 2023, for a recent review). These skills are considered fundamental in learning to read in English (Bowey, 2005; Castles et al., 2018). In general, research has shown that teachers (both pre-service and in-service) have a rather limited knowledge of these concepts. In one of the pioneering studies, Moats (1994) developed and administered the Informal Survey of Linguistic Knowledge to 89 in-service educators, including reading teachers, special education teachers, and speech-language pathologists. The survey assessed their knowledge of phonological awareness, phonics, and morphology. Moats found that in-service teachers had a limited understanding of the terminology associated with these concepts and difficulties in their practical application, such as correctly identifying the number of phonemes or morphemes in words. This limited knowledge is concerning given that current service models in schools such as the Response to Intervention (RtI) model (Fletcher & Vaughn, 2009) emphasize the importance of delivering high-quality whole classroom instruction as a first step towards the prevention of reading difficulties.

Subsequent studies across different English-speaking countries replicated Moats’ (1994) findings, highlighting teachers’ difficulties in accurately defining key terms such as phonological awareness or in identifying the correct number of phonemes in words (e.g., Bos et al., 2001; Brady et al., 2009; Dahl-Leonard et al., 2025; Fielding-Barnsley, 2010; Mather et al., 2001; Moats & Foorman, 2003; Piasta et al., 2020; Pittman et al., 2020; Porter et al., 2022; Washburn et al., 2011). For example, in a study with 1369 in-service teachers, Porter et al. (2022) found that they could answer correctly only 73% of the phonological awareness questions and 64% of the phonics questions. Teachers have also been found to struggle determining the correct number of morphemes in words (e.g., Moats, 1994; Porter et al., 2022; Washburn et al., 2011) with the percentage of correct answers hovering around 35% (about half of the percentage of correct answers reported for phonological awareness or phonics questions).

Previous research on the connection between teachers’ knowledge of language and literacy concepts and students’ reading outcomes has produced mixed findings (e.g., Carlisle et al., 2011; Hudson, 2022; Lane et al., 2008; Piasta et al., 2009, 2020; Porter et al., 2024; Podhajski et al., 2009; Spear-Swerling & Brucker, 2004). On the one hand, some studies have reported positive associations between teachers’ knowledge (most often measured with surveys) and students’ reading-related skills (most often measured with direct assessments) (e.g., Lane et al., 2008; Podhajski et al., 2009; Porter et al., 2024). For example, in a study with 512 kindergarten and Grade 1 teachers, Porter et al. (2024) showed that teachers’ knowledge of foundational literacy skills (measured with a 50-item survey from McMahan et al., 2019) predicted their Spring scores in Foundational Skills (a cluster of concepts that includes basic features of print, phonological awareness, and the ability to apply grade-level phonics and word analysis skills in decoding words), even after controlling for student-level characteristics such as gender, ethnicity and their Fall scores in Foundational Skills. In turn, some studies have reported small or no effects (e.g., Carlisle et al., 2009, 2011; Hudson, 2022; Piasta et al., 2009, 2020). For example, working with a group of 42 first-grade teachers, Piasta et al. (2009) found no significant effects of teachers’ knowledge on their students’ growth in word identification, after controlling for their earlier word identification scores.

There might be four reasons for these inconsistent findings: First, whereas in Porter et al.’s (2024) study the outcome measure was a cluster of different concepts, in Piasta et al.’s (2009) study, the outcome was just a word identification task. Because the teachers’ knowledge score usually represents their total knowledge of different concepts, it has better chances to predict a cluster score made up of different concepts than a score that is based on a single concept. Second, it may be due to the various limitations of the existing studies. For example, some studies have focused exclusively on the students’ reading outcomes without considering students’ initial level in reading (e.g., Hudson, 2022; Podhajski et al., 2009). Controlling for students’ initial level in reading is considered a rather conservative test as there is often very little variance left in the dependent variable (e.g., students’ reading performance in the Spring) for other variables such as teachers’ knowledge to explain. Third, several studies in this field examined the role of professional development in teachers’ knowledge of language and literacy concepts (e.g., McCutchen et al., 2002; Podhajski et al., 2009; Spear-Swerling & Brucker, 2004) and performed group comparisons to test if students of teachers in the experimental group (i.e., those teachers who received training) performed better than students of teachers in the control group. Although improved teachers’ knowledge in the experimental group was often accompanied by a significant improvement in their students’ reading scores (e.g., Spear-Swerling & Brucker, 2004), this does not answer the question whether teachers’ knowledge of language and literacy concepts explains individual differences in students’ reading outcomes. Finally, when researchers conducted the first couple of studies connecting teachers’ knowledge of language and literacy concepts to their students’ reading outcomes (e.g., McCutchen et al., 2002; Podhajski et al., 2009), they were constrained by the analytic strategies that were available at that time. That forced researchers to either aggregate student data to the classroom level or ignore the nonindependence of teachers’ knowledge scores to their students’ reading scores.

### 1.2. Teachers’ Perceived Ability to Teach Reading

An area that has also attracted researchers’ interest is that of teachers’ perceived ability to teach reading, often referred to as self-efficacy in reading instruction. Teachers’ self-efficacy beliefs are based on Bandura’s (1986) social cognitive theory, which describes teachers’ beliefs in their ability to succeed in specific situations. According to this theory, to provide effective instruction, teachers need to believe they have the necessary knowledge and skills to influence their students’ performance. Research has shown that teachers who feel confident in their ability to teach reading are more likely to employ effective instructional strategies, persist with struggling readers, and foster positive reading attitudes in students (e.g., Aro & Björn, 2016; Caprara et al., 2006; Forgie et al., 2022; Guo et al., 2012; Tschannen-Moran & Hoy, 2001). For example, in a longitudinal study on Italian primary school teachers’ self-efficacy and their students’ academic performance, Caprara et al. (2006) found that teachers’ self-efficacy predicted students’ reading achievement over time, even after controlling for students’ initial ability levels.

An issue that remains unclear is the connection between teachers’ perceived ability to teach reading and their knowledge of different literacy concepts. Teachers’ perceived ability to teach reading may determine the type of professional development they may pursue as well as the level of engagement in professional development that aims to increase their knowledge. The training study conducted by Podhajski et al. (2009) showed that teachers who received training on different literacy concepts (e.g., phonemic awareness, phonics) not only improved in their knowledge but also gained greater confidence in their instruction. However, there is also evidence of a weak relationship between the two. For example, Aro and Björn (2016) reported nonsignificant correlations between teachers’ perceived ability to teach different literacy concepts and their knowledge score (*r* = 0.08 with their phonology and phonics score and *r* = 0.02 with their morphology score). Thus, more research is needed on this topic.

### 1.3. The Joint Effects of Teachers’ Knowledge and Perceived Ability to Teach Reading on Students’ Reading Performance

Only a handful of studies have examined the joint effects of teachers’ perceived ability to teach reading and their knowledge of language and literacy concepts on students’ reading growth and have produced mixed findings (McCutchen et al., 2002, 2009; Parrila et al., 2024; Podhajski et al., 2009). For example, in a training study with 42 second-grade teachers, McCutchen et al. (2009) found that teachers scoring in the top quartile for both knowledge and efficacy had students with 1.5 times greater end-of-year reading gains than those in the bottom quartiles. In contrast, working with a group of 79 first-grade teachers, Parrila et al. (2024) found no direct or indirect (via quality of instruction or differentiation of instruction) effects of either teachers’ knowledge or teachers’ perceived ability on students’ decoding skills. Notably, the studies that have examined the joint effects of teachers’ knowledge and perceived ability to teach reading were all conducted with early elementary grade (often first- and second-grade) teachers and their students. Thus, it remains unclear if the two variables would predict students’ growth in reading in upper elementary grades.

### 1.4. The Present Study

The purpose of this study was to examine the role of teachers’ knowledge of language and literacy concepts and their perceived ability to teach reading in their students’ reading growth. We further examined if the effects of these variables were mediated by teachers’ perceived ability to differentiate instruction for their students. More knowledgeable teachers should be better able to differentiate instruction and this, in turn, should predict students’ reading growth (Parrila et al., 2024). We also controlled for students’ earlier reading performance (i.e., the autoregressor) and teachers’ years of teaching experience. This allowed us to examine if teachers’ knowledge and perceived ability to teach reading contribute to students’ reading growth beyond what can be explained by their earlier performance levels.

## 2. Materials and Methods

### 2.1. Participants

The participants of this study were 32 language arts teachers from seven schools in British Columbia, Canada, and their 582 English-speaking Grade 3 to 9 students (48% female; *M*_age_ = 10.6 years). Both teachers and students were recruited on a voluntary basis (participation rate was about 70% from both). Students were invited to participate in the study only if their teacher had agreed to participate by providing their written consent. The teachers were mostly female (82%), had, on average, 16.58 years of teaching experience, and the vast majority held a bachelor’s degree (93%). Seventy-one percent of the students were White, 6% Asian, 5% Hispanic, 9% First Nations Métis and Inuit (FNMI), and 9% other. This ethnicity distribution is typical of the student population in British Columbia (Statistics Canada, 2016). In addition, children came mostly from middle-income families (based on teacher reports and the location of the schools) and did not have any severe intellectual, behavioral, or sensory difficulties. Although we did not collect information on the socioeconomic status of each child, the socioeconomic status index of the participating schools (Quintile 3) shows that they were at the “average” level of socioeconomic standing. Parental consent was obtained prior to accessing students’ scores for this project. Ethics permission for the study was obtained from the University of Alberta (Approval # was Pro00065133).

### 2.2. Measures

Measures for Teachers. Teachers were administered a survey that included questions about (1) their years of teaching experience; (2) their perceived ability to teach different reading-related skills; (3) their perceived ability to differentiate instruction in reading; and (4) their knowledge of language and literacy concepts (i.e., phonological awareness, phonics, and morphology).

*Years of teaching experience*. We asked teachers to write down the number of years they had been teaching.

*Perceived ability in teaching different reading-related skills.* We asked teachers to rate from 1 (minimal) to 4 (expert) their ability to teach different aspects of literacy (e.g., *How would you rate your ability to teach phonics?*). The items were adapted from Washburn et al. (2011). The score was the sum of the scores on five items asking teachers to rate themselves in teaching (a) reading to typically developing children; (b) reading to struggling readers; (c) phonemic awareness; (d) phonics; and (e) morphology (max = 20).

*Perceived ability to differentiate instruction*. We asked teachers to rate from 1 (minimal) to 4 (expert) their ability to differentiate instruction in reading for their students.

*Knowledge of language and literacy concepts.* We assessed their knowledge of phonological awareness, phonics, and morphology by administering a survey with 29 questions (G. Georgiou et al., 2025). Of the 29 items, 8 measured teachers’ knowledge of phonological awareness, 13 teachers’ knowledge of phonics, and 8 teachers’ knowledge of morphology. Twenty-five questions were multiple-choice questions consisting of one keyed answer and four distractors. For example, participants were asked to identify the correct definition of terms (e.g., “What is morphology?”) or select the rule that explains a language concept (e.g., “What is the rule that governs the use of ‘c’ in the initial position for /k/?”). Four questions that included a total of 21 subitems measured participants’ ability to apply their understanding to tasks specific to each domain without necessarily recognizing or selecting an explicit rule. For instance, in the phonological awareness domain, a question would prompt participants to identify the number of phonemes in a given word (e.g., ship). A participant’s score was the total number of correct responses (reported as average percentage of correct responses). Cronbach’s alpha reliability in our sample was 0.82 for the whole survey and 0.79 for the phonological awareness domain, 0.82 for the phonics domain, and 0.77 for the morphology domain, respectfully.

Measures for Students. Students’ reading performance was assessed by their teachers at the beginning and end of the school year with three measures: The Sight Word Reading Efficiency (SWRE) and Phonemic Decoding Efficiency (PDE) from the Test of Word Reading Efficiency-2 (TOWRE-2; Torgesen et al., 2012) and the Test of Silent Reading Efficiency and Comprehension (TOSREC; Wagner et al., 2010). In TOWRE-2, children were asked to read aloud as many real words (SWRE; max = 108) or pseudowords (PDE; max = 66) as possible within a 45 sec time limit. Prior to testing, children were asked to read eight words or pseudowords in a practice list to establish familiarity with the task demands. The raw scores in SWRE and PDE were converted to a scaled score following the instructions in the manual and were then combined to obtain an index (standard) score. The index score was used in all analyses. Torgesen et al. (2012) reported test–retest reliability for SWRE and PDE to range from 0.83 to 0.94. In addition, the correlation between subsequent measurement points for TOWRE-2 in our sample was 0.78. In TOSREC, children were given a booklet containing 60 sentences and were given 3 min to verify by circling Yes or No if the meaning of each sentence was true or false (e.g., *Strawberries are blue*. Yes No). Each grade has a different booklet. The raw scores were converted to standard scores following the procedures in the manual. Wagner et al. (2010) reported test–retest reliability for Grades 3 to 9 to be around 0.90. In addition, the correlation between the two measurement points in our sample was 0.73.

### 2.3. Procedures

Teachers completed the survey during one of their professional development days at the beginning of the school year. Completing the survey took approximately 20 min and each teacher completed it individually. Teachers in the participating schools also tested their students twice (October and May) on TOWRE-2 and TOSREC. TOWRE-2 was individually administered and TOSREC was group-administered. This is performed as part of the school division’s policy to screen all of their students twice a year in order to identify struggling readers. For the purpose of this project, we only accessed and used the scores of the children with a parental consent. Their scores were provided to us by their teachers, after all of their personal identifying information was removed.

### 2.4. Statistical Analysis

**Figure 1:**
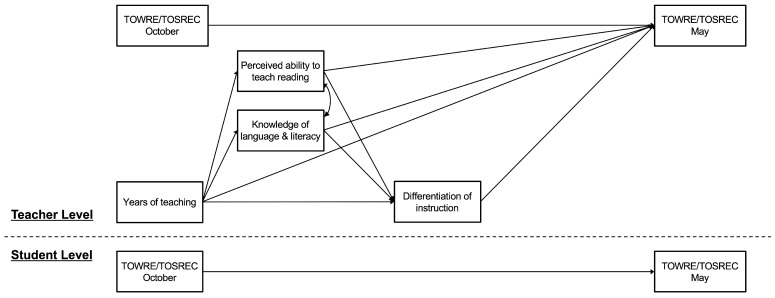
Hypothesized Model for Teachers’ Knowledge, Perceived Ability of Teaching, Differentiation of Instruction, and Students’ Reading Growth. TOWRE = Test of Word Reading Efficiency; TOSREC = Test of Silent Reading Efficiency and Comprehension.

First, descriptive statistics (including means, standard deviations, and correlations) were calculated using R (version 4.5.0; R Core Team, 2025). Next, to examine the associations among the variables at both the teacher and student levels, multilevel models were estimated separately for the two reading measures (see Figure 1). Multilevel modeling enables the differentiation of the variances in nested data into two components: variation attributable to differences between teachers and individual differences between students (Heck & Thomas, 2009). Multilevel modeling was performed using Mplus (Version 8; Muthén & Muthén, 1998–2017), and teacher variables were grand-mean centered prior to model estimation. The analysis codes are available on our Open Science Framework (OSF) project page at https://osf.io/aumex/ (see also Supplementary Material). As shown in Figure 1, the models at both levels included students’ reading scores in October (the beginning of the school year) as a predictor and those in May (the end of the school year) as the outcome. The teacher-level models also included years of experience, perceived ability to teach reading, knowledge of language and literacy concepts as predictors, as well as perceived ability to differentiate instruction as a potential mediator. Therefore, if any of the teacher variables showed a significant effect on students’ reading scores in May, it would suggest that the variable had an independent effect on students’ reading growth beyond what could be explained by the performance levels in October. Finally, to test the indirect effects of teacher variables on students’ reading scores via differentiation of instruction, mediation analysis was performed (Hayes, 2022).

All analyses handled missing data using full information maximum likelihood (FIML) estimation, which allowed the use of all observations in the dataset to estimate the model parameters (Muthén & Muthén, 1998–2017). Model fit was examined using chi-square values and three fit indices: the comparative fit index (CFI), the root-mean-square error of approximation (RMSEA), and the standardized root-mean-square residual (SRMR). Nonsignificant chi-squared values, CFI values above 0.95, RMSEA values below 0.06, and SRMR values below 0.08 indicate a good fit (Kline, 2023).

## 3. Results

### 3.1. Descriptive Analysis

Table 1 displays the descriptive statistics for all variables in the study. The teachers, on average, had about 16 years of experience (ranging from 1 to 38 years). Their differentiation of instruction was rated slightly above “moderate”. Regarding the knowledge of language and literacy concepts, the teachers had relatively strong knowledge of phonics (85.9%) and phonological awareness (75.4%), though their knowledge of morphology was relatively weaker (60.9%). Composite scores for teachers’ perceived ability to teach reading and their knowledge of language and literacy concepts were used in further analyses.

**Figure 2:**
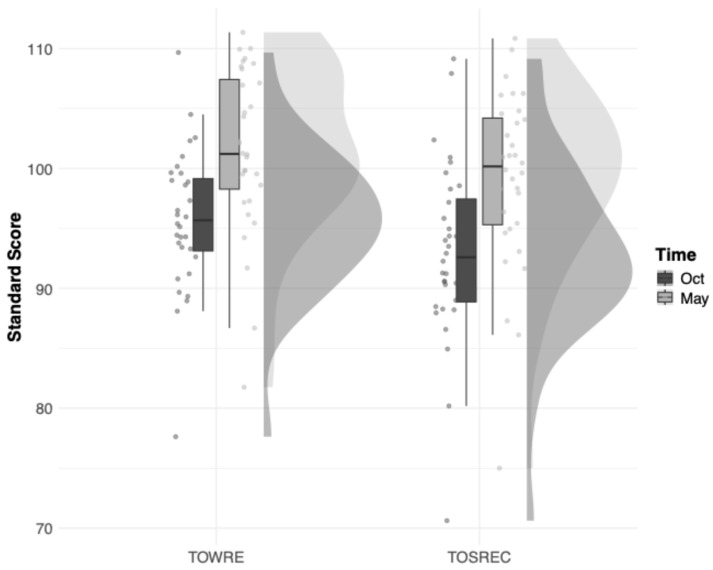
Classroom-Level Reading Scores. TOWRE = Test of Word Reading Efficiency; TOSREC = Test of Silent Reading Efficiency and Comprehension.

The means of the students’ standard scores in both reading tests indicate that, at the beginning of the school year, our sample of students in Grades 3 to 9 was reading slightly below the average (95.8 for TOWRE-2 and 93.8 for TOSREC) for what would be expected of North American children, but caught up by the end of the school year (102.1 for TOWRE-2 and 100.1 for TOSREC; see Figure 2). Intraclass correlations (ICCs) of their reading scores showed that between-classroom differences in reading skills ranged from 4.2% (TOWRE-2 in October) and 12.3% (TOSREC in October) of the total variability.

Table 2 and Table 3 display the correlations among all the variables in the study at the teacher (classroom) and student levels, respectively. As expected, students’ reading skills at the two time points were strongly correlated with each other (*r* = 0.55 to 0.85). This was also true at the classroom level: the average reading scores were strongly correlated with each other across tests and time points (*r* = 0.58 to 0.78). This indicates that both students’ and classrooms’ relative performance levels were fairly stable over time. Teachers’ years of experience and perceived ability to teach reading were moderately correlated with their perceived ability to differentiate instruction (*r* = 0.57 and 0.67, respectively). Teachers’ perceived teaching ability, but not years of teaching experience, was also moderately correlated with their knowledge of language and literacy concepts (*r* = 0.41), as well as with their students’ reading scores (*r* = 0.24 and 0.42 for TOWRE-2 and 0.38 and 0.41 for TOSREC). Additionally, teachers’ perceived ability to differentiate instruction was moderately correlated with TOWRE-2 at the end of the year (*r* = 0.44).

### 3.2. Multilevel Modeling

**Figure 3:**
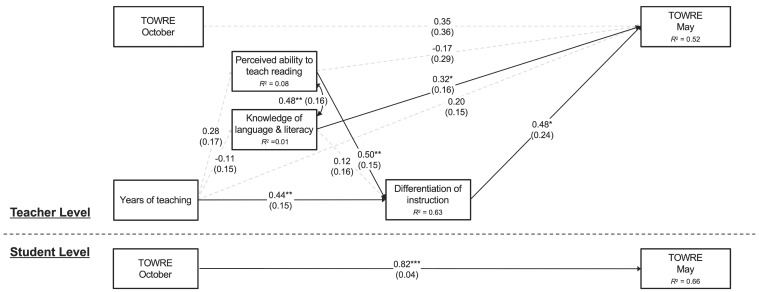
Final Model for TOWRE Scores (Standardized Estimates). Note: Numerals in parentheses indicate standard errors. * *p* < 0.05; ** *p* < 0.01; *** *p* < 0.001.

Figure 3 shows a summary of the model results for teacher variables and students’ TOWRE-2 scores. The model fit was excellent: χ^2^(4) = 2.14, *p* = 0.71, CFI = 1.00, RMSEA = 0.00, SRMR_within_ = 0.00, SRMR_between_ = 0.08. Students’ TOWRE-2 scores in October significantly predicted their TOWRE-2 scores in May at the student level (β = 0.82), but not at the teacher (classroom) level. At the teacher level, teachers’ years of experience and perceived ability to teach reading were associated with their ability to differentiate instruction (β = 0.44 and 0.50, respectively), which, in turn, predicted TOWRE-2 in May (β = 0.48). However, mediation analysis showed that the indirect effect of perceived ability to teach reading on students’ reading scores through perceived ability to differentiate instruction was not statistically significant (*p* = 0.118; see Table 4). Additionally, teachers’ knowledge about language and literacy concepts was independently associated with TOWRE-2 in May (β = 0.32) over and above the effects of the other variables.

**Figure 4:**
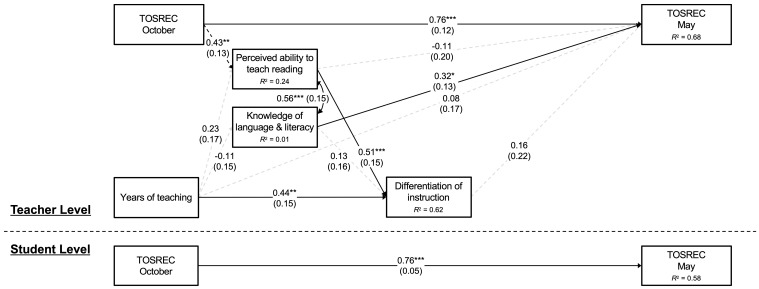
Final Model for TOSREC Scores (Standardized Estimates). Note: Numerals in parentheses indicate standard errors. The path from TOSREC in October to teachers’ perceived ability was added after the initial model estimation, and should therefore be treated as exploratory. * *p* < 0.05; ** *p* < 0.01; *** *p* < 0.001.

Figure 4 shows a summary of the final model for teacher variables and students’ TOSREC scores. An additional path from students’ TOSREC scores in October to teachers’ perceived ability to teach reading was included based on the model’s modification indices. The model fit was excellent, χ^2^(3) = 1.98, *p* = 0.58, CFI = 1.00, RMSEA = 0.00, SRMR_within_ = 0.00, SRMR_between_ = 0.06. Students’ TOSREC scores in October predicted their scores in May at both the student and teacher (classroom) levels (both β = 0.76). As in the model for TOWRE-2 reported above, teachers’ years of experience and perceived ability to teach reading were associated with their perceived ability to differentiate instruction (β = 0.44 and 0.51, respectively). For TOSREC, however, the effect of perceived ability to differentiate instruction on students’ reading scores in May did not reach statistical significance (β = 0.16, *p* = 0.475). Of the teacher variables, only teachers’ knowledge about language and literacy concepts was independently associated with TOSREC in May (β = 0.32) after controlling for the effects of the other variables. None of the indirect effects were statistically significant (Table 4).

## 4. Discussion

The purpose of this study was to examine the role of teachers’ knowledge of language and literacy concepts and of their perceived ability in teaching these concepts in students’ reading growth in Grades 3 to 9. Our findings showed first that, regardless of the reading outcome, teachers’ knowledge of language and literacy concepts had a direct effect on students’ reading performance at the end of the school year. In contrast, teachers’ perceived ability in teaching these literacy concepts did not have a significant effect on students’ reading performance. To our knowledge, this is the first study on this topic to recruit upper elementary and junior high school children and their teachers, and one of the few that examined the role of teachers’ knowledge of language and literacy concepts in students’ reading outcomes after controlling for students’ initial levels in reading (see Parrila et al., 2024; Piasta et al., 2009; Porter et al., 2024, for previous studies that used the same methodology). Including an autoregressor (i.e., children’s reading ability at an earlier point in time) changes the question from one of predicting growth in general into one of predicting further growth or “unexpected” growth that cannot be accounted for by the skill itself at an earlier time (see Parrila et al., 2004, for a discussion on the use of an autoregressor). This is particularly important in studies with upper elementary students. Because individual differences in reading are established early on in children’s school life (e.g., G. K. Georgiou et al., 2021; Khanolainen et al., 2024; Parrila et al., 2004; Wagner et al., 1997), they explain a large amount of variance in their future reading performance. If we want to see if more knowledgeable teachers make a difference in students’ reading growth that is not explained by the students’ earlier reading ability, then controlling for the effects of the autoregressor is crucial.

The direct effect of teachers’ knowledge of language and literacy concepts on students’ reading growth suggests that upper-elementary grade teachers need to have a deeper and more explicit understanding of their writing system in order to meet the demands of literacy instruction in these grade levels. The fact that our sample comprised upper-elementary grade teachers may explain the discrepancy with the findings of earlier studies with Grade 1 teachers that reported nonsignificant effects of teachers’ knowledge of language and literacy concepts on their students’ reading performance (e.g., Parrila et al., 2024; Piasta et al., 2009). It is possible that teachers with a deep understanding of their writing system are needed in upper elementary grade levels in order to make a significant contribution to students’ growth in reading over and above what is expected from their earlier reading ability. This makes sense if we consider that in these grade levels students encounter multisyllabic/multimorphemic words and teachers with better knowledge of morphology are better equipped to teach their students how to read these words efficiently. However, it is also worth noting here that compared to previous studies that measured the same areas of teachers’ content knowledge (e.g., Carlisle et al., 2009; Pittman et al., 2020; Piasta et al., 2009), our teacher participants obtained much higher scores (see Table 1). If further growth in children’s reading in upper elementary grade levels requires more knowledgeable teachers, then our sample would be better off than those of previous studies (e.g., Parrila et al., 2024; Piasta et al., 2009). This explanation is in line with Piasta et al.’s (2009) finding that for students of more knowledgeable teachers, more time in explicit instruction led to larger word-reading gains. First, as shown in Table 1, our teachers possessed relatively good knowledge of the language and literacy concepts (particularly in phonological awareness and phonics) and, second, they have been providing explicit and systematic instruction to their students (based on anecdotal information from their principals and discussion we had with our teacher participants).

Although teachers’ perceived ability to teach reading correlated significantly with students’ reading outcomes (with the exception of TOWRE-2 in October), it did not survive the statistical control of the other predictors (e.g., students’ earlier reading ability, teachers’ knowledge of language and literacy concepts). The nonsignificant effect of teachers’ perceived ability on students’ reading growth replicates Parrila et al.’s (2024) finding with Grade 1 teachers and is also in line with Klassen et al.’s (2011) meta-analytic findings showing a nonsignificant correlation between teachers’ self-efficacy and students’ reading performance. To explore whether the potential effect of teachers’ perceived ability was masked by the effect of differentiation of instruction (also measured with a self report), we performed an additional analysis without differentiation of instruction in the model. The effect of perceived ability to teach reading on students’ reading outcomes continued to be nonsignificant (β = 0.11, *p* = 0.637 for TOWRE-2; β = 0.00, *p* = 0.998 for TOSREC). Although teachers’ perceived ability significantly predicted their perceived ability to differentiate instruction and perceived ability to differentiate instruction predicted students’ growth in TOWRE-2, we did not find evidence of an indirect effect on students’ reading growth either (i.e., the mediation analysis was nonsignificant). There might be two explanations for the discrepancy between our findings and those of previous studies that found a significant effect of teachers’ self-efficacy on students’ reading performance. First, it may be due to the fact that these studies did not include teachers’ knowledge of language and literacy concepts (e.g., Guo et al., 2012) or students’ earlier reading ability (e.g., Leino et al., 2022) in the same model. Second, it may be due to differences in the performed statistical analyses. Some of the previous studies that reported an association between teachers’ self-efficacy and students’ reading outcomes relied on group comparisons and not on regression analyses or multilevel analyses that treat teachers’ self-efficacy as a continuous predictor variable to explain individual differences in students’ reading performance (as done in our study). For example, McCutchen et al. (2002) found that after providing professional development, teachers’ knowledge and self-efficacy in teaching reading improved significantly more than that of a control group that did not receive training. They also found that the reading performance of the students of teachers in the experimental group improved significantly more than that of the control group. On the basis of this, McCutchen et al. (2002) concluded that increased teachers’ self-efficacy was related to improved students’ reading performance. Obviously, this is different from using teachers’ perceived ability in a model with other variables to explain individual differences in students’ reading performance.

The nonsignificant effect of teachers’ perceived ability in our study may also suggest that the effects of this variable on students’ reading growth are more nuanced and mediated by factors such as teaching quality or work atmosphere in classroom (see Piasta et al., 2020), both of which were not assessed in this study. It is also possible that teachers’ perceived ability to teach reading may be a consequence rather than a cause of students’ reading outcomes, as suggested by the significant effect of TOSREC in October on teachers’ perceived ability in our sample. Finally, it is possible that there is a misalignment between what teachers think they are good at and what actually helps children improve in their reading ability.

### 4.1. Implications for Practice

Our findings have some important practical implications. First, they suggest that school authorities should try to improve their teachers’ knowledge of language and literacy concepts as they directly impact their students’ reading growth. More knowledgeable teachers can design better and more targeted activities for their students, which leads to better student outcomes. Obviously, teacher training is a big issue and involves multiple stakeholders including post-secondary institutions and teacher associations, but it is an issue that warrants our immediate attention. We already have several examples of training studies consistently showing that after training teachers on evidence-based practices and providing coaching opportunities, there is a measurable improvement in students’ reading performance (e.g., McCutchen et al., 2009; Podhajski et al., 2009). This also aligns with evidence from mixed method studies in which teachers who were able to move the needle in their students’ literacy indicated that professional development within their schools played a catalytic role in improving their knowledge, which was then put into use when teaching their students (G. K. Georgiou et al., 2020; Kierstead et al., 2023).

In addition, teachers’ self-efficacy is something we need to pay closer attention to. We already know that teachers who are more confident in their ability to teach reading are more likely to employ effective instructional strategies, persist with struggling readers, and foster positive reading attitudes in their students (e.g., Guo et al., 2012; Tschannen-Moran & Hoy, 2001). Our study further showed that teachers’ perceived ability to teach different literacy concepts was positively and significantly related to their knowledge of language and literacy concepts (*r* = 0.41) and to their perceived ability to differentiate instruction (*r* = 0.67). We can build teachers’ self-efficacy in teaching reading by engaging them in high-quality professional development on evidence-aligned structured literacy approaches, by building professional learning communities where teachers can learn from colleagues with strong literacy instructional skills, and by giving them access to effective literacy resources (e.g., G. K. Georgiou et al., 2020; Tschannen-Moran & McMaster, 2009).

### 4.2. Limitations and Future Research

Some limitations of the present study are worth noting. First, our survey assessed teachers’ knowledge only in phonological awareness, phonics and morphology, and this may have narrowed our coverage. Our decision was influenced by two factors: First, adding vocabulary and comprehension questions would make our survey very long and from our experience teachers are reluctant to fill out long surveys. Second, our students’ outcome measures tap into sight word reading and decoding efficiency that are more influenced by phonological awareness, phonics and morphology than by vocabulary. Thus, we believe leaving out vocabulary and comprehension questions did not have a significant impact on our results. However, we acknowledge that having some questions on vocabulary and reading comprehension would probably give us a fuller picture of teachers’ knowledge. Second, we did not measure teaching quality and as a result we could not examine if the effects of teachers’ knowledge and perceived ability are mediated by teaching quality (see Connor et al., 2009, for some evidence on the mediating role of teaching quality). Third, we measured teachers’ content knowledge with a focus on language and literacy concepts and not pedagogical content knowledge. Again, we made this decision because we tried to keep the time to complete our survey as reasonable as possible. A future study should examine how both content and pedagogical content knowledge contribute to students’ reading growth. Fourth, our study covered only one school year and we measured teachers’ knowledge of language and literacy concepts only once. A future study should follow children and their teachers over multiple years and explore if students’ earlier reading performance also influences teachers’ knowledge of language and literacy concepts in the future. Fifth, teachers rated their own ability to teach different reading skills and to differentiate instruction, and this may not accurately reflect their true ability. As shown by Cunningham et al. (2004), teachers often overestimate their knowledge and ability to teach, and this may have attenuated the correlations with student outcomes. In our decision to use a self-report measure, we took into account teachers’ sensitivity around being observed while teaching. Finally, teachers’ ability to differentiate instruction was measured with a single item. Future studies should replicate our findings using more items.

## 5. Conclusions

The findings of this study add to a small but growing number of studies examining the role of teachers’ knowledge of language and literacy concepts and perceived ability in teaching reading in their students’ reading growth (e.g., Carlisle et al., 2009; McCutchen et al., 2009; Parrila et al., 2024; Porter et al., 2024; Piasta et al., 2009) by showing that only teachers’ knowledge was a significant predictor and its influence on students’ reading growth were direct. This highlights the need to develop the conditions within each school that will allow teachers to acquire an explicit understanding of the foundational literacy skills that are critical in learning to read in English.

## Figures and Tables

**Table 1:** Descriptive Statistics for Teacher- and Child-Level Variables.

Variables	n	Mean	SD	Min	Max	Skew	Kurtosis	ICC [95% CI]
Teacher-level variables								
Years of teaching	30	16.58	12.30	1	38	0.07	−1.61	–
Ability to teach typically developing readers	32	2.62	0.55	1	3	−1.04	−0.03	–
Ability to teach struggling readers	32	2.44	0.76	1	4	−0.44	−0.65	–
Ability to teach phonemic awareness	32	2.53	0.76	1	4	−0.31	−0.45	–
Ability to teach phonics	32	2.62	0.61	2	4	0.36	−0.82	–
Ability to teach morphological awareness	32	2.47	0.72	1	4	−0.40	−0.49	–
Ability to differentiate instruction for students	32	2.50	0.67	1	4	−0.31	−0.40	–
Knowledge of phonological awareness	32	75.42	17.80	13.33	100	−1.45	2.44	–
Knowledge of phonics	32	85.94	14.11	56.25	100	−0.56	−1.09	–
Knowledge of morphology	32	60.94	17.39	25	93.75	−0.15	−0.75	–
Student-level variables								
TOWRE-2 October	582	95.80	16.50	53	139	−0.27	0.03	0.042 [0.007, 0.108]
TOWRE-2 May	579	102.05	17.14	53	147	−0.15	−0.08	0.091 [0.041, 0.182]
TOSREC October	569	93.79	17.13	54	143	−0.11	−0.15	0.123 [0.063, 0.227]
TOSREC May	580	100.09	16.83	54	146	−0.03	0.07	0.103 [0.049, 0.199]

*Note*: TOWRE = Test of Word Reading Efficiency; TOSREC = Test of Silent Reading Efficiency and Comprehension.

**Table 2:** Correlations Between the Variables in the Study (Teacher-Level).

Variables	1.	2.	3.	4.	5.	6.	7.
1. Years of teaching							
2. Perceived ability to teach reading	0.28						
3. Differentiation of instruction	0.57 **	0.67 **					
4. Knowledge of language and literacy	−0.11	0.41 *	0.28				
5. TOWRE-2 October	0.08	0.24	−0.01	0.04			
6. TOWRE-2 May	0.34	0.42 *	0.44	0.28	0.62 **		
7. TOSREC October	0.13	0.38 *	0.14	−0.06	0.60 **	0.62 **	
8. TOSREC May	0.19	0.41 *	0.27	0.18	0.66 **	0.74 **	0.73 **

Note: TOWRE = Test of Word Reading Efficiency; TOSREC = Test of Silent Reading Efficiency and Comprehension. * *p* < 0.05; ** *p* < 0.01.

**Table 3:** Correlations Between the Variables in the Study (Student-Level).

Variables	1.	2.	3.
1. TOWRE-2 October			
2. TOWRE-2 May	0.78 **		
3. TOSREC October	0.58 **	0.58 **	
4. TOSREC May	0.67 **	0.68 **	0.78 **

Note: TOWRE = Test of Word Reading Efficiency; TOSREC = Test of Silent Reading Efficiency and Comprehension. ** *p* < 0.01.

**Table 4:** Indirect Effects of Teachers’ Perceived Ability and Knowledge on Students’ Reading Scores (Unstandardized Estimates).

Indirect Effect	Estimate	*SE*	*p*
Perceived ability → Differentiation → TOWRE	0.073	0.046	0.118
Perceived ability → Differentiation → TOSREC	0.161	0.233	0.489
Knowledge → Differentiation → TOWRE	0.019	0.029	0.504
Knowledge → Differentiation → TOSREC	0.009	0.011	0.409

Note: TOWRE = Test of Word Reading Efficiency; TOSREC = Test of Silent Reading Efficiency and Comprehension.

## Data Availability

Those interested can request our data from the corresponding author.

## Supplementary Materials

The analysis codes are available on our Open Science Framework (OSF) project page at https://osf.io/aumex/.

## References

[B1-behavsci-15-01408] Aro M., Björn P. M. (2016). Preservice and inservice teachers’ knowledge of language constructs in Finland. Annals of Dyslexia.

[B2-behavsci-15-01408] Bandura A. (1986). Social foundations of thought and action: A social cognitive theory.

[B3-behavsci-15-01408] Bos C., Mather N., Dickson S., Podhajski B., Chard D. (2001). Perceptions and knowledge of preservice and inservice educators about early reading instruction. Annals of Dyslexia.

[B4-behavsci-15-01408] Bowey J. A., Snowling M. J., Hulme C. (2005). Predicting individual differences in learning to read. The science of reading: A handbook.

[B5-behavsci-15-01408] Brady S., Gillis M., Smith T., Lavalette M., Liss-Bronstein L., Lowe E., North W., Russo E., Wilder T. D. (2009). First grade teachers’ knowledge of phonological awareness and code concepts: Examining gains from an intensive form of professional development and corresponding teacher attitudes. Reading and Writing: An Interdisciplinary Journal.

[B6-behavsci-15-01408] Caprara G. V., Barbaranelli C., Steca P., Malone P. S. (2006). Teachers’ self-efficacy beliefs as determinants of job satisfaction and students’ academic achievement: A study at the school level. Journal of School Psychology.

[B7-behavsci-15-01408] Carlisle J. F., Correnti R., Phelps G., Zeng J. (2009). Exploration of the contribution of teachers’ knowledge about reading to their students’ improvement in reading. Reading and Writing: An Interdisciplinary Journal.

[B8-behavsci-15-01408] Carlisle J. F., Kelcey B., Rowan B., Phelps G. (2011). Teachers’ knowledge about early reading: Effects on students’ gains in reading achievement. Journal of Research on Educational Effectiveness.

[B9-behavsci-15-01408] Castles A., Rastle K., Nation K. (2018). Ending the reading wars: Reading acquisition from novice to expert. Psychological Science in the Public Interest.

[B10-behavsci-15-01408] Connor C. M., Piasta S. B., Fishman B., Glasney S., Schatschneider C., Crowe E., Underwood P., Morrison F. J. (2009). Individualizing student instruction precisely: Effects of child x instruction interactions on first graders’ literacy development. Child Development.

[B11-behavsci-15-01408] Cunningham A. E., Firestone A. R., Zegers M., Cabell S. Q., Neuman S. B., Terry N. P. (2023). Measuring and improving teachers’ knowledge in early literacy. Handbook on the science of early literacy.

[B12-behavsci-15-01408] Cunningham A. E., Perry K. E., Stanovich K. E., Stanovich P. J. (2004). Disciplinary knowledge of K-3 teachers and their knowledge calibration in the domain of early literacy. Annals of Dyslexia.

[B13-behavsci-15-01408] Dahl-Leonard K., Hall C., DeCoster J., Solari E. (2025). Elementary educator self-efficacy and knowledge to teach reading. Teachers and Teaching.

[B14-behavsci-15-01408] Desimone L. M. (2009). Improving impact studies of teachers’ professional development: Toward better conceptualizations and measures. Educational Researcher.

[B15-behavsci-15-01408] Desimone L. M., Garet M. S. (2015). Best practices in teachers’ professional development in the United States. Psychology, Society & Education.

[B16-behavsci-15-01408] Fielding-Barnsley R. (2010). Australian pre-service teachers’ knowledge of phonemic awareness and phonics in the process of learning to read. Australian Journal of Learning Difficulties.

[B17-behavsci-15-01408] Fletcher J. M., Vaughn S. (2009). Response to intervention: Preventing and remediating academic difficulties. Child Development Perspectives.

[B18-behavsci-15-01408] Forgie J. C., Hu J., Boccalon M. (2022). Pre-service and in-service early childhood educators’ self-efficacy and knowledge for early literacy instruction. Cogent Education.

[B21-behavsci-15-01408] Georgiou G., Peyton A., Antoniuk A., Beach P., Fraser A., Kirby J. R., Klein P., Li G., Metsala J., Mousavi A., Nickel J., Savage R., Joshi R. M. (2025). Pre-service teachers’ knowledge of language and literacy concepts: The skeleton in Canada’s closet?. Annals of Dyslexia.

[B19-behavsci-15-01408] Georgiou G. K., Inoue T., Papadopoulos T. C., Parrila R. (2021). Examining the growth trajectories and cognitive predictors of reading in a consistent orthography: Evidence from a 10-year longitudinal study. Applied Psycholinguistics.

[B20-behavsci-15-01408] Georgiou G. K., Kushnir G., Parrila R. (2020). Moving the needle on literacy: Lessons learned from a school where literacy rates have improved over time. Alberta Journal of Educational Research.

[B22-behavsci-15-01408] Guo Y., Connor C. M., Yang Y., Roehrig A. D., Morrison F. J. (2012). The effects of teacher qualification, teacher self-efficacy, and classroom practices on fifth graders’ literacy outcomes. The Elementary School Journal.

[B23-behavsci-15-01408] Hattie J. (2023). Visible learning: The sequel: A synthesis of over 2100 meta-analyses relating to achievement.

[B24-behavsci-15-01408] Hayes A. F. (2022). Introduction to mediation, moderation, and conditional process analysis: A regression-based approach.

[B25-behavsci-15-01408] Heck R. H., Thomas S. L. (2009). An introduction to multilevel modeling techniques.

[B26-behavsci-15-01408] Hudson A. K. (2022). Upper elementary teachers’ knowledge of reading comprehension, classroom practice, and student’s performance in reading comprehension. Reading Research Quarterly.

[B27-behavsci-15-01408] Joshi R. M., Binks E., Hougen M., Dahlgren M. E., Ocker-Dean E., Smith D. L. (2009). Why elementary teachers might be inadequately prepared to teach reading. Journal of Learning Disabilities.

[B28-behavsci-15-01408] Kang H. S., Cha J., Ha B.-W. (2013). What should we consider in teachers’ professional development impact studies? Based on the conceptual framework of Desimone. Creative Education.

[B29-behavsci-15-01408] Khanolainen D., Psyridou M., Eklund K., Aro T., Torppa M. (2024). Predicting reading fluency growth from grade 2 to age 23 with parental and child factors. Scientific Studies of Reading.

[B30-behavsci-15-01408] Kierstead M., Georgiou G., Poth C., Mack E. (2023). Effective school literacy culture and learning outcomes: The multifaceted leadership role of the principal. Alberta Journal of Educational Research.

[B31-behavsci-15-01408] Klassen R. M., Tze V. M., Betts S. M., Gordon K. A. (2011). Teacher efficacy research 1998–2009: Signs of progress or unfulfilled promise?. Educational Psychology Review.

[B32-behavsci-15-01408] Kline R. B. (2023). Principles and practice of structural equation modelling.

[B33-behavsci-15-01408] Lane H. B., Hudson R. F., Leite W. L., Kosanovich M. L., Strout M. T., Fenty N. S., Wright T. L. (2008). Teachers’ knowledge about reading fluency and indicators of students’ fluency growth in reading first schools. Reading & Writing Quarterly.

[B34-behavsci-15-01408] Leino K., Nissinen K., Sirén M. (2022). Associations between teacher quality, instructional quality and student reading outcomes in Nordic PIRLS 2016 data. Large-Scale Assessments in Education.

[B35-behavsci-15-01408] Mather N., Bos C., Babur N. (2001). Perceptions and knowledge of preservice and inservice teachers about early literacy instruction. Journal of Learning Disabilities.

[B36-behavsci-15-01408] McCutchen D., Abbott R. D., Green L. B., Beretvas S. N., Cox S., Potter N. S., Quiroga T., Gray A. L. (2002). Beginning literacy: Links among teacher knowledge, teacher practice, and student learning. Journal of Learning Disabilities.

[B37-behavsci-15-01408] McCutchen D., Green L., Abbott R. D., Sanders E. A. (2009). Further evidence for teacher knowledge: Supporting struggling readers in grades three through five. Reading and Writing.

[B38-behavsci-15-01408] McMahan K. M., Oslund E. L., Odegard T. N. (2019). Characterizing the knowledge of educators receiving training in systematic literacy instruction. Annals of Dyslexia.

[B39-behavsci-15-01408] Moats L. C. (1994). The missing foundation in teacher education: Knowledge of the structure of spoken and written language. Annals of Dyslexia.

[B40-behavsci-15-01408] Moats L. C., Foorman B. R. (2003). Measuring teachers’ content knowledge of language and reading. Annals of Dyslexia.

[B41-behavsci-15-01408] Muthén L. K., Muthén B. O. (1998–2017). Mplus user’s guide.

[B42-behavsci-15-01408] Nippold M. A. (2018). The literate lexicon in adolescents: Monitoring the use and understanding of morphologically complex words. Perspectives of the ASHA Special Interest Groups.

[B43-behavsci-15-01408] O’Dea M., Harris J. (2019). Effectiveness of reflective practice in a TA peer-mentorship program. Canadian Engineering Education Association (CEEA).

[B44-behavsci-15-01408] Parrila R., Inoue T., Dunn K., Savage R., Georgiou G. (2024). Connecting teachers’ language knowledge, perceived ability and instructional practices to Grade 1 students’ literacy outcomes. Reading and Writing: An Interdisciplinary Journal.

[B45-behavsci-15-01408] Parrila R., Kirby J. R., McQuarrie L. (2004). Articulation rate, naming speed, verbal short-term memory, and phonological awareness: Longitudinal predictors of early reading development?. Scientific Studies of Reading.

[B46-behavsci-15-01408] Piasta S. B., Connor C. M., Fishman B. J., Morrison F. J. (2009). Teachers’ knowledge of literacy concepts, classroom practices, and student reading growth. Scientific Studies of Reading.

[B47-behavsci-15-01408] Piasta S. B., Park S., Farley K. S., Justice L. M., O’Connell A. A. (2020). Early childhood educators’ knowledge about language and literacy: Associations with practice and children’s learning. Dyslexia.

[B48-behavsci-15-01408] Pittman R. T., Zhang S., Binks-Cantrell E., Hudson A., Joshi R. M. (2020). Teachers’ knowledge about language constructs related to literacy skills and student achievement in low socio-economic status schools. Dyslexia.

[B49-behavsci-15-01408] Podhajski B., Mather N., Nathan J., Sammons J. (2009). Professional development in scientifically based reading instruction: Teacher knowledge and reading outcomes. Journal of Learning Disabilities.

[B50-behavsci-15-01408] Porter S. B., Odegard T. N., Farris E. A., Oslund E. L. (2024). Effects of teacher knowledge of early reading on students’ gains in reading foundational skills and comprehension. Reading and Writing: An Interdisciplinary Journal.

[B51-behavsci-15-01408] Porter S. B., Odegard T. N., McMahan M., Farris E. A. (2022). Characterizing the knowledge of educators across the tiers of instructional support. Annals of Dyslexia.

[B52-behavsci-15-01408] R Core Team (2025). R: A language and environment for statistical computing.

[B53-behavsci-15-01408] Spear-Swerling L., Brucker P. O. (2004). Preparing novice teachers to develop basic reading and spelling skills in children. Annals of Dyslexia.

[B54-behavsci-15-01408] Statistics Canada (2016). Just the facts: A portrait of the school-age population and measures of student achievement.

[B55-behavsci-15-01408] Torgesen J. K., Wagner R. K., Rashotte C. A. (2012). Test of word reading efficiency—Second edition (TOWRE-2).

[B56-behavsci-15-01408] Tschannen-Moran M., Hoy A. W. (2001). Teacher efficacy: Capturing an elusive construct. Teaching and Teacher Education.

[B57-behavsci-15-01408] Tschannen-Moran M., McMaster P. (2009). Sources of self-efficacy: Four professional development formats and their relationship to self-efficacy and implementation of a new teaching strategy. The Elementary School Journal.

[B58-behavsci-15-01408] Wagner R. K., Torgesen J. K., Rashotte C. A., Hecht S. A., Barker T. A., Burgess S. R., Donahue J., Garon T. (1997). Changing relations between phonological processing abilities and word-level reading as children develop from beginning to skilled readers: A 5-year longitudinal study. Developmental Psychology.

[B59-behavsci-15-01408] Wagner R. K., Torgesen J. K., Rashotte C. A., Pearson N. A. (2010). Test of silent reading efficiency and comprehension (TOSREC).

[B60-behavsci-15-01408] Washburn E. K., Joshi R. M., Binks-Cantrell E. S. (2011). Teacher knowledge of basic language concepts and dyslexia. Dyslexia.

